# Recent advances in immunotherapy and molecular targeted therapy for gastric cancer

**DOI:** 10.2144/fsoa-2023-0002

**Published:** 2023-03-17

**Authors:** Yuri Yoshinami, Hirokazu Shoji

**Affiliations:** 1 Department of Gastrointestinal Medical Oncology, National Cancer Center Hospital, 5-1-1 Tsukiji, Chuo-ku, Tokyo, 104-0045, Japan

**Keywords:** anti-PD-1 or PD-L1 inhibitors, gastric cancer, immune checkpoint inhibitors, immunotherapy, molecular targeted therapy

## Abstract

Our increasing understanding of the molecular biological characteristics of cancer and of cancer genomics is facilitating the development of immunotherapy and molecular targeted drugs for gastric cancer. After the approval of immune checkpoint inhibitors (ICIs) for melanoma in 2010, many different cancers have been shown to respond to such treatments. Thus, the anti-PD-1 antibody nivolumab was reported to prolong survival in 2017, and ICIs have become the mainstay of treatment development. Many clinical trials of combination therapies with cytotoxic agents and molecular-targeted agents, as well as combinations of immunotherapeutic agents acting via different mechanisms, are currently underway for each treatment line. As a result, further improvements in therapeutic outcomes for gastric cancer are anticipated in the near future.

## Plain language summary

Gastric cancer is the fourth most common malignant tumor and also ranks fourth as a cause of death from cancer. However, even with chemotherapy, prognosis is limited to 12–15 months.

The recent development of immune checkpoint inhibitors (ICIs) encourages optimism that these may represent novel standards-of-care for AGC, with clear clinical benefits. Many clinical trials of combination therapies with cytotoxic agents and molecular-targeted agents, as well as combinations of immunotherapeutic agents, are currently underway for each treatment line. As a result, further improvements in therapeutic outcomes for gastric cancer are anticipated in the near future.

**Figure 1.**
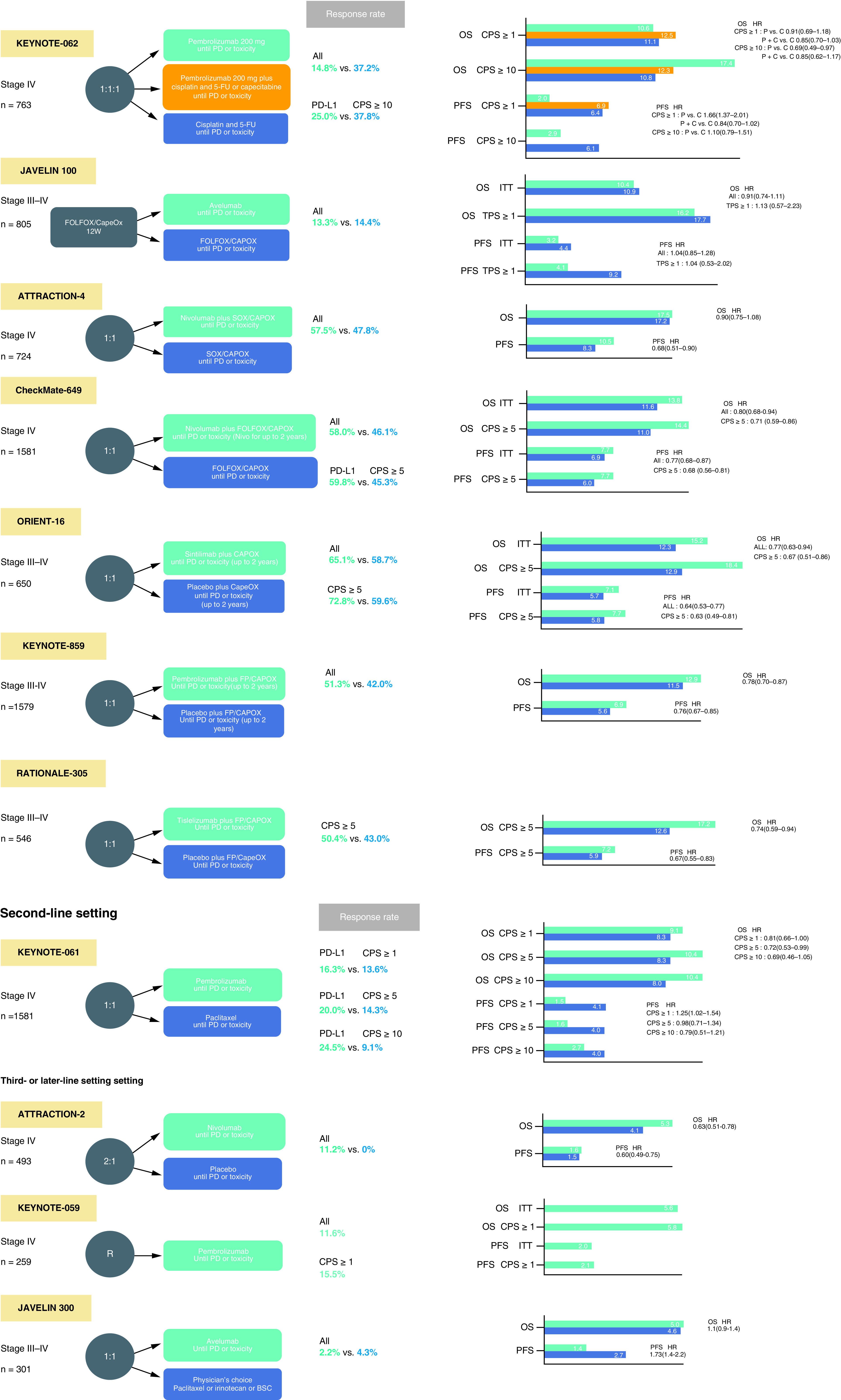
Overview and the results of important gastric cancer clinical trials. CAPOX: Capecitabine + oxaliplatin; CPS: Combined positive score; FOLFOX: 5-fluorouracil + leucovorin + oxaliplatin; FP: 5-fluorouracil + Cisplatin; Nivo: Nivolumab; OS: Overall survival; PFS: Progression-free survival; PD: Progressive disease; SOX: tegafur/gimeracil/oteracil potassium + oxaliplatin.

Globally, gastric cancer is the fourth most common malignant tumor and also ranks fourth as a cause of death from cancer [1]. Despite the establishment of first-line combination therapies containing a fluoropyrimidine and a platinum agent (supplemented with the anti-HER2 antibody trastuzumab for patients with cancer over-expressing HER2), and second-line paclitaxel with or without ramucirumab, as standard treatments for advanced unresectable or recurrent gastric cancer (AGC), median survival is only 12–15 months. The recent development of immune checkpoint inhibitors (ICIs) encourages optimism that these agents may represent novel standards-of-care for AGC, with clear clinical benefits reported for some patient cohorts (Figure 1) [2,3]. For AGC, the humanized anti-programmed cell death-1 (PD-1) IgG4 monoclonal antibody pembrolizumab (pembro) is licensed as a second-line or later therapy for tumors with high microsatellite instability (MSI-H) or those with a high mutation rate (Tumor mutational burden-high:TMB-H) [4]. A combination of pembro and trastuzumab as first-line therapy for HER2-over-expressing AGC has been approved by the US Food and Drug Administration (FDA). A survival benefit on third-line or subsequent therapy using a different anti-PD-1 antibody, nivolumab (nivo) in an Asian patient population has also been reported; notably, this was independent of the expression of the PD-1 ligand PD-L1 by the tumor in the ATTRACTION-2 trial [5]. Nivo was also effective for first-line therapy in combination with standard cytotoxic agents in CheckMate-649 [6]. Hence, it is encouraging to note potential clinical benefits in several trials of experimental immunotherapies in AGC; these also include combinations of different ICIs, such as anti-PD-1 together with anti-CTLA-4 antibodies, or ICIs and different targeted agents, as well as chimeric antigen receptor T (CAR-T) cell therapies. The present review focused on the current status of immunotherapies and molecular targeted treatments for gastric cancer.

## Molecular characterization & immunological signatures in gastric cancer

Four subtypes of gastric cancer have been proposed, based on a 2014 Cancer Genome Atlas (TCGA) study. These were those associated with infection with Epstein–Barr virus (EBV), those with microsatellite instability (MSI), those with chromosomal instability (CIN) and genomically stable cancers (GS). Differential diagnosis of these subtypes relied somatic copy number variations, whole-exome sequencing (WES), DNA methylation profiling, mRNA sequencing (RNAseq), microRNA sequencing, and reverse-phase protein array investigations [7]. We will now describe and discuss these four subtypes separately.

### Epstein–Barr virus

Genetic evidence of EBV infection is present in 10% of gastric cancers [8]. Consistent with this, a 100% objective response rate (ORR) was recorded in a phase II study of pembro treatment of EBV-positive AGC, albeit only in 6 patients [9]. Also, Panda *et al.* [10] reported a durable response to therapy with avelumab, an anti-PD-L1 antibody, despite the fact that EBV+ AGC has a low TMB. However, this was a case report of just one patient, but a phase Ib/II study of 4 patients recorded an ORR of 25% to therapy with a different anti-PD-1 antibody, toripalimab [11]. Taking these few results together, it must be noted that at the present time the effectiveness of single-agent anti-PD-1 for EBV+ AGC remains open to question, and more trials will be required with more patients in order to come to a definitive conclusion.

### Microsatellite instability

Tumors with a high degree of microsatellite instability (MSI-H tumors) have a high TMB as a result, as well as displaying hypermethylation. These abnormalities lead to the presence of larger amounts and different types of neoantigens on the cancer cells [10]. It is believed that MSI-H tumors are commonly infiltrated by large numbers of CD8^+^ T cells because they recognize multiple neoantigens expressed by the cancer cells due to the higher TMB. Correspondingly, immune checkpoint molecule expression, including PD-L1, is increased in the tumor microenvironment (TME) [12]. In line with these findings, as well as several different clinical trials suggesting that durable responses could be expected, pembro has been approved by the FDA for treating patients with previously-treated MSI-H/MMR-D (mismatch repair-deficient) solid tumors, including AGC [4,13]. Accordingly, pembro has also been approved in Japan for treating patients with previously-treated MSI-H solid tumors of any type.

### Chromosomal instability

Tumors with chromosomal instability (CIN) are often found at the gastroesophageal junction/cardia. A common characteristic of CIN tumors is frequent TP53 mutation and a relatively high degree of amplification of genes encoding receptor tyrosine kinases (RTKs) [7].

### Genomic stability

About 20% of gastric cancers exhibit a stable genome (GS tumors). They include a high frequency of diffuse-type tumors with major somatic alterations to the genome, namely *CDH1*, *ARIDIA*, and *RHOA*. They commonly present with a recurring inter-chromosomal translocation involved in cell motility [7].

### Comparison of gastric cancer subtypes

EBV-positive or MMR-D AGC tumors each account for 6.2% of cases in pathologic stage IV disease [14]. As alluded to above, these two subtypes exhibit different immunological profiles compatible with a higher propensity to respond to treatment with ICIs. MSI-H/MMR-D AGC patients experience poorer progression-free survival (PFS) when their first-line treatment consists of cytotoxic chemotherapy, but they can achieve durable clinical responses on later treatment with anti-PD-1 antibodies [15]. In contrast, most CIN tumors appear to prevent T cells and CD68^+^ macrophages from entering the TME. These findings provide a rationale for approaches that target immunosuppressive macrophages or other suppressive mechanisms in order to facilitate the activity of ICIs for treating GC. There may also be utility in employing the so-called “PD-L1 combined positive score” (CPS). The CPS is currently employed to determine whether ICI therapy is appropriate in several malignancies such as AGC [6,16–18]. The utility of this approach for predicting clinical ICI efficacy in AGC will be considered next.

## Treatments targeting PD-1/PD-L1

### Results of first-line therapies

The KEYNOTE-062 phase III trial compared first-line pembro as monotherapy or pembro + chemotherapy with chemotherapy alone for AGC tumors with PD-L1 CPS ≥1 or CPS ≥10 [18]. In terms of OS, it was concluded that pembro was not less effective than chemotherapy for patients with CPS ≥1 tumors (median OS 10.6 vs 11.1 months; HR: 0.91 [99.2% CI: 0.69–1.18], p = 0.162, non-inferiority margin = 1.2), but there was a tendency toward worse median PFS (2.0 vs 6.4 months) and a poorer ORR (15% vs 37%) relative to chemotherapy. Thus, pembro monotherapy is certainly not more effective than chemotherapy for tumors with CPS ≥1. However, it did tend to prolong OS relative to chemotherapy when CPS was ≥10 (median 17.4 vs 10.8 months; HR: 0.69; 95% CI: 0.49–0.97, not statistically significant). Moreover, pembro + chemotherapy did not result in better OS compared with chemotherapy in tumors with either CPS ≥1 (12.5 vs 11.1 months; HR: 0.85; 95% CI: 0.70–1.03; p = 0.05) or CPS ≥10 (12.3 vs 10.8 months; HR: 0.85; 95% CI: 0.62–1.17; p = 0.16). Regarding toxicity, grade 3–5 treatment-related adverse events (TRAEs) with pembro alone, pembro + chemotherapy, and chemotherapy alone were 17%, 73% and 69%, respectively.

Unfortunately, the JAVELIN 100 phase III trial of avelumab also failed to meet its primary objective and did not reveal any sign of improved OS for AGC patients on avelumab maintenance versus continued chemotherapy or best supportive care as first-line treatment, either in the whole patient cohort (median 10.4 vs 10.9 months; HR: 0.91; p = 0.1779) or even in those whose tumors were PD-L1+ (i.e., >1% of tumor cells) (median 16.2 vs 17.7 months; HR: 1.13; p = 0.6352) [19]. Nonetheless, when defined by CPS >1 patients with PD-L1-positive tumors did show a trend toward better OS in the avelumab arm (median 14.9 vs 11.6 months; HR: 0.72).

CheckMate-649, a randomized, open-label, comparative study carried out at 175 sites in 29 countries and regions, including Japan, set out to compare effectiveness and safety of nivo + chemotherapy with chemotherapy alone [6]. At the time this trial was begun, it initially had two arms, namely ipi + nivo and chemotherapy, but as the latter combination was expected to be effective, the design was altered to a 3-armed trial with nivo + chemotherapy. However, the ipi + nivo arm was stopped on June 5, 2018, based on the advice of the independent data monitoring committee taking into consideration the incidence of AEs and deaths from disease progression. Compared with chemotherapy alone, nivo + chemotherapy yielded more significant improvements in OS (HR: 0.71 [98.4% CI: 0.59–0.86]; p < 0.0001) and PFS (HR: 0.68 [98% CI: 0.56–0.81]; p < 0.0001) in patients whose tumors expressed PD-L1 with a CPS ≥5 (on minimum follow-up of 12.1 months). It was also noted that there were significant improvements in PFS and OS even in patients with a PD-L1 CPS ≥1 as well as in the entire cohort. These outcomes resulted in the approval of this combination as a first-line treatment for AGC, regardless of tumor PD-L1 levels, in several countries including Japan. Also at 24 months follow-up, nivo + chemotherapy continued to achieve better OS than chemotherapy alone in patients whose tumor PD-L1 CPS was ≥5 (median OS: 14.4 months (95% CI: 13.1–16.2) versus 11.1 months (10.0–12.1), respectively). However, nivo + ipi versus chemotherapy for tumors with PD-L1 CPS ≥5 failed to meet the prespecified significance cut-off (median OS: 11.2 months (95% CI: 9.2–13.4) versus 11.6 (95% CI: 10.1–12.7); HR: 0.89; 96.5% CI: 0.71–1.10; p = 0.2302 [20].

Nonetheless, the efficacy of FOLFOX chemotherapy + nivo and ipi was suggested by data presented at ESMO 2022 [21]. Thus, AIO-STO-0417 (Moonlight) is a four-armed investigator-initiated trial, of which the results of two arms (A1/A2) were presented at ESMO 2022. Here, 90 patients were randomized 1:2 to FOLFOX + nivo 240 mg; q2w and ipi 1 mg/kg; q6w administered in parallel (arm A1) or three cycles of FOLFOX induction treatment followed by nivo and ipi (arm A2). The primary end point was PFS at 6 months, and the main secondary end points were OS, PFS, ORR and safety. It was reported that 6-month PFS was 57% in arm A1 versus 28% in A2 (p = 0.012) with a median PFS of 8.4 versus 4.0 months, respectively (p = 0.006). Median OS had not been reached in A1 but was 9.1 months in A2, and the ORR was 47% versus 30%, with similar results in those with PD-L1+ tumors.

Another study, ATTRACTION-4, a randomized, open-label study (Part 1) has evaluated tolerability and safety of nivo together with SOX or CAPOX conducted at 13 sites in Japan and one other country. A randomized, double-blind, comparative study (Part 2) to assess the efficiency and safety of nivo with placebo together with chemotherapy (SOX or CAPOX) was carried out at 130 sites in 3 countries or regions including Japan [22]. With regard to efficacy in part 2, the interim analysis of PFS, which was one of the primary end points, verified the superiority of nivo + chemotherapy over chemotherapy alone (median 10.45 vs 8.34 months; HR: 0.68; p = 0.0007). However, another primary end point, OS, was not improved by nivo + chemotherapy relative to chemotherapy alone (median 17.45 vs 17.15 months; HR: 0.90; p = 0.257).

The percentage of patients who received posttreatment in ATTRACTION-4 was 65.3%. For the treatment of patients with AGC, it has been reported that survival after disease progression is prolonged in a higher proportion of patients who received posttreatment after disease progression [23]. The larger fraction of patients receiving post-treatment in ATTRACTION-4 than in CheckMate-649 may have contributed to the failure to achieve a statistically significant OS benefit of nivo + chemotherapy relative to chemotherapy only. In addition, PTX/RAM [24] is recommended as second-line therapy for patients with AGC in Japan and overseas. In addition, nivo monotherapy is approved in eight countries or regions including Japan. All of these treatments have been shown to be effective in prolonging OS, and the use of post-treatment therapies including nivo alone is thought to have made it difficult to detect the effect on OS prolongation of nivo + chemotherapy as first-line treatment.

In addition, development of Chinese ICI trials has been remarkable. The phase III ORIENT-16 placebo-controlled trial of the anti-PD-1 antibody sintilimab + chemotherapy in 650 patients with advanced adenocarcinoma of the stomach showed that the combination was superior to chemotherapy + placebo for OS in all randomized patients (median OS 15.2 vs 12.3 months, HR: 0.77; 95% CI: 0.63–0.94, p = 0.0090). When OS was assessed separately for the 61% of patients whose tumors were PD-L1 CPS ≥5, median OS was 18.4 versus 2.9 months (HR: 0.66; 95% CI: 0.51–0.86, p = 0.0023) [25].

Most recently, the results of the KEYNOTE-859 study were reported [26]. This is a randomized, double-blind, phase III trial comparing pembro + chemotherapy to placebo + chemotherapy as primary treatment for patients with HER2-negative AGC. The primary end point is OS, and secondary end points include PFS, ORR, duration of response (DOR), and safety. The study randomized 1,579 enrolled patients to receive pembrolizumab (200 mg every 3 weeks for up to ~2 years) + fluoropyrimidine and platinum-based chemotherapy or placebo + chemotherapy. Compared with placebo, pembro + chemotherapy yielded more significant improvements in OS (median OS: 12.9 months (95% CI: 11.9–14.0) versus.11.5 months (10.6–12.1), HR: 0.78 [95% CI: 0.70–0.87]; p < 0.0001) and PFS (median PFS: 6.9 months (95% CI: 16.3–7.2) versus 5.6 months (95% CI: 5.5–5.7), HR: 0.76 [95% CI: 0.67–0.85]; p < 0.0001) in ITT population. In terms of toxicity, the all-grade TRAEs were 96% in the pembro + chemotherapy group, 94% in the placebo group. The respective rates of grade 3 or higher events were 59% and 51%. Immune-related adverse events tended to be more common in the pembro + chemotherapy group and included hypothyroidism (15.3%), hyperthyroidism (5.6%) and colitis (3.3%).

An interim analysis of RATIONALE-305 trial was reported in February 2023 [27]. It was a global, double-blind, phase III. In this trial, 546 patients with HER2-negative AGC or EGC were randomized to receive tislelizumab (TIS, 200 mg every 3 weeks) + chemotherapy (CAPOX or FP) or placebo + chemotherapy as first-line treatment. The patients were included regardless of PD-1 status and were assigned 1:1 to the TIS group or the placebo group. The primary end point was OS in PD-L1 positive (PD-L1 score ≥5% b) and ITT analysis set. The results of the PD-L1 positive population were reported in this issue. Median OS was 17.2 months (95% CI: 13.9–21.3) in the TIS group versus 12.6 months (95% CI: 12.0–14.4) in the placebo group and was significantly longer in the TIS group (HR: 0.74, 95% CI: 0.59–0.94, p = 0.0056). PFS was 7.2 months (95% CI: 5.8–8.4) in the TIS group and 5.9 months (95% CI: 5.6–7.0) in the placebo group (HR: 0.67, 95% CI: 0.55–0.83). The response rate was 50.4% (95% CI: 44.3–56.4) in the TIS group and 43.0% (95% CI: 37.1–49.1) in the placebo group. TRAEs leading to treatment discontinuation were more common in the TIS group than in the placebo group (22.4% vs 12.1%), but the incidence of grade 3 or higher adverse events (64.7% vs 62.9%), serious adverse events (42.3% vs 36.8%), and death (8.8% vs 7.7%) were similar between the two treatment groups. These data suggest that this treatment is a new first-line option. Additional reports are expected.

### Second-line therapies

KEYNOTE-061, a phase III trial of second-line pembro for AGC with PD-L1 CPS ≥1 failed to show any significant prolongation of OS (median 9.1 vs 8.3 months; HR: 0.82) relative to paclitaxel [16]. OS curves crossed each other near the median, with paclitaxel tending to be more favorable up to 8 months, after which pembro was better than paclitaxel. In this this trial, patients with PDL-1-negative tumors tended to have a shorter OS with pembro than paclitaxel. Hence, patient selection is important to determine who should receive pembro as second-line treatment, emphasized by the finding that long-term survival benefit with pembro in this trial was in fact reported in several subgroups of patients, such as those with an ECOG PS of 0, PD-L1 CPS ≥10 and MSI-H tumors. Importantly, median OS was not reached with pembro whereas it was 8.1 months with paclitaxel (HR: 0.42) in a post-hoc analysis of MSI-H patients. Kaplan-Meier plots confirmed a durable clinical benefit after 2 years, with better 24-month OS rates achieved using pembro treatment relative to paclitaxel (19.9% vs 8.5%). Importantly, the superiority of pembro over paclitaxel became more pronounced as the level of PD-L1 expression increased (i.e., CPS ≥5, +15.4%; CPS ≥10, +21.3%). This suggests the potential usefulness of CPS to select patients most likely to benefit from pembro treatment [28].

### First approvals in AGC: third- & later-line therapy

In the phase III ATTRACTION-2 trial, nivo treatment was reported to result in improved OS relative to placebo for AGC patients after ≥2 previous lines of chemotherapy (median OS 5.26 vs 4.14 months; HR: 0.63; p < 0.0001) [5]. PFS (median 1.61 vs 1.45 months; HR ≥0.60; p < 0.0001) and ORR (11.2%- vs 0%; p < 0.0001) were also better on nivo treatment. A lack of relationship between survival benefit and PD-L1 expression by the tumor cells is suggested by these results, albeit tumor samples were available from <40% of patients. TRAEs of any grade were recorded in 43% of nivo-treated patients, 10% of which were grade 3 or 4 events. The type of all-grade TRAEs in at least 5% of patients treated with nivo were pruritus (9%), diarrhea (7%), rash (6%), and fatigue (5%). The most common grade 3 or 4 TRAEs were decreased appetite, diarrhea, fatigue, and increased aspartate transaminase. Nivo was approved in Asia, including in Japan, following the release of the results of the ATTRACTION-2 trial.

The phase II trial KEYNOTE-059 include an evaluation of the safety and effectiveness of pembro as a third-line or subsequent treatment for AGC [17]. The ORR was 11.6% (95% CI: 8.0–16.1%) in the whole cohort, and 15.5% for patients with PD-L1-positive tumors (CPS ≥1), but only 6.4% for PD-L1-negative tumors (CPS <1). Median PFS was 2.0 months (95% CI: 2.0–2.1), and 6-month PFS was 14.1% (95% CI: 10.1–19.7%). Median OS was 5.6 months (95% CI: 4.3–6.9), and 6-month OS was 46.5% (95% CI: 40.2–52.6%). The safety data for pembro in this trial were similar to nivo in ATTRACTION-2. TRAEs of any grade occurred in 60% of patients, with 18% being grade 3 or 4. However, the phase III trial JAVELIN 300 did not indicate that administration of the anti-PD-L1 antibody avelumab resulted in an OS improvement in AGC patients relative to investigator-selected third-line chemotherapy including paclitaxel or irinotecan (median OS 4.6 vs 5.0 months; HR: 1.1; p = 0.81) [29].

### Perioperative therapy

Many clinical data have now accumulated documenting the effectiveness of anti-PD1 antibody therapy either as a single agent or together with standard chemotherapy in the neoadjuvant setting to treat patients with triple-negative breast cancer, non-small-cell lung cancer, melanoma, or head and neck cancer [30–33]. The phase II NEONIPIGA trial evaluated neoadjuvant nivo + ipilimumab (ipi) as well as nivo in the adjuvant setting for localized MMR-D/MSI-H GC or esophagogastric adenocarcinomas (EGC) [34]. The primary end point, pathological CR, was reported to be nearly 60% (90% CI: 41.8–74.1%).

Pembro treatment in the perioperative setting for EGC is being assessed in KEYNOTE-585, an international double-blind phase III trial aiming for enrollment of 860 patients [35]. Patients receive pembro postoperatively with additional chemotherapy (cisplatin + capecitabine/5-FU) compared 1:1 with patients receiving placebo + chemotherapy. Another trial, the MATTERHORN trial, an international double-blind placebo-controlled phase III study aiming to recruit 900 patients with resectable EGC, is evaluating OS of the anti-PD-L1 antibody durvalumab in the perioperative setting. The patients are treated whether or not their tumors over-express PD-L1 and also receive FLOT (5-fluorouracil, folinic acid, oxaliplatin, docetaxel) in comparison with FLOT alone [36]. In the adjuvant setting, the phase III trial ATTRACTION-5 is currently ongoing to compare standard chemotherapy (S-1 or capecitabine + oxaliplatin) together with nivo for pathological stage III GC (including esophagogastric junction cancer) after D2 or more extensive lymph node dissection. However, at the time of writing, perioperative ICI for GC is only being administered in controlled clinical trials.

### Responses of tumors with high levels of DNA mismatch repair deficiency or high-stage microsatellite instability

Proof of principle that DNA mismatch repair deficiency (dMMR) cancers are particularly susceptible to treatment by inhibition of the PD-L1/PD-1 interaction was first demonstrated in a trial of pembro for dMMR colorectal cancer. Subsequently, it was established that immunotherapy with ICIs is effective in a subset of patients with dMMR tumors, regardless of site of origin or histology. However, the frequency of microsatellite instability (MSI-H)-high stage IV AGC in Japan is low, only about 6.7% [37]. The phase II KEYNOTE-158 trial enrolled 24 GC patients and reported that 11 (46%) achieved an objective response, including 4 complete responses (CRs), with a median PFS of 11 months [38]. Subsequently, post-hoc analyses of data from KEYNOTE-059 (pembro as third-line therapy), KEYNOTE-061 (pembro as second-line vs chemotherapy), and KEYNOTE-062 (pembro first-line therapy, with pembro + chemotherapy vs chemotherapy alone) indicated that patients with dMMR/MSI-H tumors had superior survival, relative to the overall study population, no matter at which treatment line [39].

In subgroup analysis of CheckMate-649, there was an advantage in terms of OS for patients with MSI-H tumors treated with nivo + chemotherapy relative to chemotherapy alone (unstratified HR: 0.38; 95% CI: 0.17–0.84) [20]. Similarly, nivo + ipi treatment resulted in better median OS (unstratified HR: 0.28; 95% CI: 0.08–0.92).

In the perioperative setting, nivo + ipi was evaluated in the single-arm GERCOR NEONIPIGA study for locally advanced resectable dMMR/MSI-H GC patients in Europe (n = 32). The primary end point was pathologic CR, a high rate of which as reported (58.6%; 90% CI: 41.8 to 74.1). Meanwhile, a phase II single-arm study, WJOG13320G, is underway to assess the effectiveness of nivo + ipi as first-line treatment for Japanese patients with unresectable advanced or recurrent or metastatic MSI-H GC [40].

### Impact of a high tumor mutation burden

It seems that 5–19% of gastric adenocarcinomas have a high TMB [41,42], although this differs from patient to patient. High TMB tumors do still have less mutations than dMMR tumors, but TMB appears to be an independent biomarker of benefit for ICI immunotherapy. Based on the results of KEYNOTE-158 (which did not include any patients with advanced GC), pembro is now approved for any solid tumor, including advanced GC, provided that they have a TMB ≥10 mutations per megabase (mut/Mb), and have progressed on standard regimens. However, validation studies were conducted primarily in lung and urothelial cancers, and since TMB thresholds are likely to vary by tumor type, it is unclear whether this is an appropriate threshold to define high TMB, especially in GC without MSI [43].

## Combining anti-PD-1 with anti-CTLA4 antibodies

Nivo and the cytotoxic T-lymphocyte antigen-4 (CTLA-4)-specific antibody ipi have different mechanisms of action that contribute to the release of anti-tumor T-cells from checkpoint blockade and to the induction of *de novo* anti-tumor T-cell responses, respectively [44]. A common finding emerging from phase III GC trials combining chemotherapy and immunotherapy is the difference in efficacy depending on the level of PD-L1 expression. For example, in Checkmate-649 there was a lack of benefit when AGCs expressed little PD-L1 (HR: 0.92, 95% CI: 0.70–1.23 and 0.94, 95% CI: 0.78–1.13 for CPS <1 and <5, respectively). A better understanding of the reasons for different outcomes depending on the level of tumor PD-L1 in AGC is required. Currently, a phase III trial (ATTRACTION-6), of nivo + 1 mg/kg ipi + chemotherapy, relative to chemotherapy alone, is running in some Asian countries (Table 1).

**Table 1.** This table shows ongoing pivotal clinical trials of anti-PD-1/PD-L1 therapies for gastric cancer.

Clinical trial	Phase	Line	Region	Patient selection	Regimen	Primary end point	Result
Attraction-6	III	1	Global	All (HER2-)	Nivo + Ipi + SOX/CAPOX SOX/CAPOX	OS	Ongoing
MAHOGANY	II/III	1	Global	HER2+	Margetuximab + Retifanlimab Margetuximab + Retifanlimab + CAPOX/FOLFOX Margetuximab + Tebotelimab + CAPOX/FOLFOX Margetuximab + CAPOX/FOLFOX Tmab + CAPOX/FOLFOX	OS	Ongoing
LEAP-015	III	1	Global	All (HER2-)	Pembo + Lenva + CAPOX/FOLFOX CAPOX/FOLFOX	OS/PFS	Ongoing

CAPOX: Capecitabine + oxaliplatin; FOLFOX: 5-FU + leucovorin + oxaliplatin; Ipi: Ipilimumab; Lenva: Lenvatinib; Nivo: Nivolumab; OS: Overall survival; PFS: Progression-free survival; Pembro: Pembrolizumab; SOX: Tegafur/gimeracil/oteracil potassium + oxaliplatin; Tmab: Trastuzumab.

## Anti-PD-1 antibody combined with multikinase inhibitors

Inhibition of the VEGF pathway has been reported to block cancer progression in preclinical studies and reduce the accumulation of immunosuppressive cells in the tumor, such as regulatory T cells (Tregs), TAMs and myeloid-derived suppressor cells, at the same time as increasing mature dendritic cell (DC) infiltration [45]. VEGF receptor multikinase inhibitors, as well as other receptor tyrosine kinases, such as regorafenib(rego) or lenvatinib, were reported to decrease immunosuppressive cells and enhance the antitumor activity of PD-1 blockade in animal models [46,47]. Moreover, the presence of immunosuppressive cells has been associated with rapid progression during checkpoint blockade, resulting in hyperprogressive disease (HPD). Thus, it has been proposed that targeting these immunosuppressive cells with inhibitors of different kinases may reduce HPD and increase the anti-tumor activity of checkpoint blockade. A phase Ib trial in AGC of rego + nivo achieved an ORR of 44% and median PFS of 5.6 months [48]. Importantly, 3 of 7 AGC patients who had proven refractory to earlier anti-PD-1 treatment did respond to rego + nivo, supporting the notion that resistance to anti-PD-1 treatment can indeed be overcome by rego. Based on these results, the phase III INTEGRATE IIb trial comparing rego + nivo to chemotherapy is undergoing (ClinicalTrials.gov Identifier: NCT0487936). There has also been a Phase II trial of lenvatinib + pembro for AGC patients in a first- or second-line setting, yielding encouraging results (69% ORR with a median PFS of 7.1 months) [49]. This ORR is remarkably high considering the expected response rate to pembro monotherapy is 15% and <5% for lenvatinib. These results suggest robust potential synergies for such combined treatments for AGC. The results of the safety run-in phase (part 1) of LEAP015, (a two-part, phase III study of safety and efficacy of lenvatinib + pembro + chemotherapy), yielded a high preliminary ORR of 73% and DCR, 93% [50].

## Anti-PD-1 antibody together with HER2-targeted treatments

Trastuzumab has been reported to increase HER2 internalization and cross-presentation by DCs, and to upregulate PD-1 and PD-L1 expression, facilitate tumor-infiltrating lymphocyte ingress, and modulate MHC class II antigen expression, all of which enhanced HER2-specific T-cell responses in preclinical models [51]. A phase II trial of the addition of trastuzumab + pembro to first-line chemotherapy yielded encouraging results (ORR 91%, median PFS 13.0 months) [52]. Subsequently, the phase III KEYNOTE-811 trial of pembro + trastuzumab and chemotherapy yielded a significant 22.7% improvement of the ORR in patients receiving pembro rather than placebo (77.4 vs 51.9%, p = 0.00006) [53]. CRs were also more frequent on pembro treatment (11.3 vs 3.1%). Moreover, patients receiving pembro experienced superior responses (median change from baseline, 65 vs 49%; ≥80% decrease from baseline, 32.3 vs 14.8%). Grade 3 or 4 AEs were recorded in 57.1% of patients on pembro and in 57.4% of those receiving the placebo. These interim results of KEYNOTE-811 led to accelerated FDA approval for adding pembro to trastuzumab and chemotherapy for first-line treatment of HER2-positive AGC. Unlike in KEYNOTE-811, a combination of nivo with trastuzumab and chemotherapy assessed in the Ni-High study reported no safety issues but with a similar ORR of 76.2% (95% CI: 60.6–86.9), DCR of 97.6% and 6-month PFS of 68.7% (95% CI: 51.7–81.3) [54].

Trastuzumab deruxtecan (T-DXd), an antibody-drug conjugate comprising an anti-HER2 antibody, a topoisomerase I inhibitor, and a cleavable linker, has been reported to improve the ORR and OS of patients with HER2-positive AGC previously treated with trastuzumab-containing chemotherapy relative to third-line or later-line [55]. T-DXd was also assessed in the single-arm DESTINY-Gastric02 trial as a second-line treatment for patients with centrally confirmed HER2-positivity in the U.S. and Europe who progressed on a regimen containing trastuzumab (n = 79). The primary end point was ORR confirmed by an independent central review. This was achieved in 38% of the patients recruited (95% CI: 27.3–49.6), with CRs occurring in 3 patients. Median PFS was 5.5 months (95% CI: 4.2–7.3) [56].

An ongoing phase Ib/II trial, DESTINY-Gastric03, is assessing antitumor activity of T-DXd alone or in combination with chemotherapy and/or immunotherapy (NCT04379596). Consistent with the above results, a Phase II trial of margetuximab (an Fc-optimized, anti-HER2 monoclonal antibody with increased affinity) combined with pembro in HER2 IHC3+ AGC reported encouraging results, with an ORR of 24% and DCR of 62% in a second-line setting [57]. The Phase II/III MAHOGANY trial, assessing the efficacy of margetuximab with INCMGA00012 (anti-PD-1 antibody) or MGD013 (anti-PD-1/anti-LAG3 antibody) + chemotherapy in previously untreated AGC patients is underway [NCT04082364]. Most recently, in the MAHOGANY trial Cohort A, margetuximab together with INCMGA00012 yielded an ORR of 52.4% [58], warranting further investigation in the ongoing trial (Table 1).

Zanidatamab is a bispecific antibody targeting two different HER2 epitopes, namely, extracellular domain 4 (including the Trastuzumab binding site) and extracellular domain 2 (with the Pertuzumab binding site). Results from a phase II trial documented that zanidatamab together with chemotherapy (mFOLFOX6, CAPOX, or FP) yielded promising results (ORR 68.2%, DCR 90.9%, and a median response duration of 16.4 months) [59]. Due to these results, a phase III trial is currently underway to test zanidatamab + chemotherapy (CAPOX or FP) + the anti-PD-1 antibody tislelizumab as first-line therapy for HER2-positive AGC (NCT04276493).

Tucatinib is a highly selective small molecule TKI acting on HER2, possessing a 1000-fold greater degree of selectivity for HER2 relative to the closely related receptor EGFR. This therefore minimizes the risk of EGFR-related toxicity caused by dual HER2/EGFR inhibition, according to cell signaling assays [60]. In a phase II study of 22 colorectal cancer patients who were refractory to chemotherapy, treatment with a combination of tucatinib and trastuzumab resulted in an ORR of 55%. A phase I/II clinical trial of this combination is currently underway for HER2-positive GC, also to investigate the safety of trastuzumab and other anticancer agents (i.e., pembro, FOLFOX, CAPOX) + tucatinib (NCT04430738).

## Anti-PD-1 antibody together with Claudin 18.2 (CLDN18.2)-targeted treatments

Claudin 18.2 (CLDN18.2) is expressed in diverse human cancers and is the dominant isoform in gastric and pancreatic cancer. Zolbetuximab is a first-in-class chimeric monoclonal immunoglobulin G1 antibody that binds to CLDN18.2 and mediates tumor cell death via antibody-dependent cellular cytotoxicity (ADCC) and complement-dependent cytotoxicity (CDC) [61]. In the phase IIb FAST study, zolbetuximab + chemotherapy significantly improved survival compared with chemotherapy alone in AGC patients expressing CLDN18.2 [62]. The phase III SPOTLIGHT placebo-controlled trial of zolbetuximab + chemotherapy in 565 patients with CLDN18.2-positive, HER2-negative AGC showed that the combination was superior to chemotherapy + placebo for PFS and OS in all randomized patients (median PFS 10.61 vs 8.67 months, HR: 0.751; 95% CI: 0.589–0.942, p = 0.0066; median OS 18.23 vs 15.54 months, HR: 0.75; 95% CI: 0.601–0.936, p = 0.0053) [63]. Zolbetuximab is being investigated in combination with immunotherapeutic agents in the ILUSTRO trial in patients with CLDN18.2 positive AGC (NCT03505320).

## ICIs for other immune checkpoints

As well as PD-1 and CTLA-4, several other immune checkpoints are known to modulate cytotoxic T-cells, including lymphocyte activation gene-3 (LAG3) and T-cell immunoreceptor with immunoglobulin and ITIM domains (TIGIT).

### Lymphocyte activation gene-3 (LAG3)

As the name suggests, the cell surface molecule LAG3, structurally similar to CD4, is expressed by activated CD4^+^ and CD8^+^ T cells, and its modulates their activity. It therefore offers a potential target for cancer immunotherapy. Like CD4, LAG-3 is a receptor for MHC class II molecules that are present in large amounts on antigen-presenting cells but may also be aberrantly expressed by cancer cells. Because LAG-3 ligation triggers a negative regulatory signal, this can result in immune tolerance and cancer immune escape [64]. The PD-1/PD-L1 intracellular signaling pathways are different from those triggered by binding of LAG3 to its specific ligands. It has been proposed that blockade of both PD-1 and LAG3 together may act synergistically to restore T cell function in cancer. From results of the RELATIVITY-047 trial, the anti-LAG3 antibody relatlimab was approved for combination immunotherapy together with nivo for malignant melanoma. There is a current phase II trial ongoing to evaluate the efficacy and safety of a combination of relatlimab, nivo and chemotherapy in GC (NCT03662659).

### T-cell immunoreceptor with immunoglobulin & ITIM domains (TIGIT)

TIGIT is an immune checkpoint receptor present on cytotoxic T cells, memory T cells, Tregs and NK cells [65]. The ligation of TIGIT with either of its two ligands, CD155 or CD112, suppresses cytotoxic T cell and NK cell activation. In contrast, TIGIT expression by Tregs seems to amplify their immunosuppressive functions. Cancer cells utilize the TIGIT pathway to evade immunosurveillance, and anti-TIGIT antibodies may amplify immune responses by inhibiting its binding to CD155 and CD112. Hence, there has been much interest in developing anti-TIGIT antibodies. From the results of a cohort of GC patients in the KEYVIBE-001 trial, a phase 1 trial is being conducted in patients with solid tumors. Treatments with the anti-TIGIT antibody vibostolimab together with pembro have yielded potentially encouraging results [66]. The ORR for patients on vibostolimab 200 mg + pembro was 13%, median DOR was 10 months, and median PFS was 2 months (95% CI: 2–4). Based on these data, a combination of vibostolimab, pembro and chemotherapy is being investigated as a first-line treatment for GC in the phase III KEYVIBE-001 trial, ongoing in several different cancer types.

### Bispecific antibodies

A bispecific antibody (BsAb) is an antibody that can bind to two individual antigens simultaneously. It thus has dual specificity, unlike common monoclonal antibodies, and because a single agent is responsible for the function of two different drugs it may theoretically be superior to ordinary monoclonal antibodies.

BiTE (bispecific T-cell engager) antibodies are constructed to bind both to T cells and also to antigens expressed on tumors and can activate the anti-cancer cytotoxic potential of the patient's own T cells [67]. Thus, when BiTE molecules engage both cytotoxic T cells and tumor cells, the T cells are activated to clonally expand, resulting in the rapid production of large numbers of specific effector cells and enhancing the efficacy of BiTE therapy. Numerous clinical trials of BsAb have been carried out, but so far, they are only approved for hematologic malignancies [68]. For GC, AMG910, a BiTE BsAb that activates T cells in cancer cells expressing the target antigen CLDN18.2 is currently being assessed in a phase I study (NCT04260191). In addition, a phase I study of ASP2138, a bispecific antibody targeting CLDN 18.2 and CD3, is ongoing (NCT05365581).

### Chimeric antigen receptor T cells (CAR-T)

Although this approach to cell therapy has achieved remarkable clinical effectiveness in different hematologic malignancies [69,70], it has thus far achieved very limited success in treating solid tumors [71]. However, there are at least 30 clinical trials of CAR-T cells ongoing for treating GC. Of these, the interim results of a phase I clinical trial of CLDN18.2-targeted CAR-T cells (CT041) yielded an ORR of 57.1% and 75.0% DCR with a 6-month OS rate of 81.2% and acceptable tolerability in CLDN18.2-positive GC patients [72].

## Conclusion

**Figure 2.**
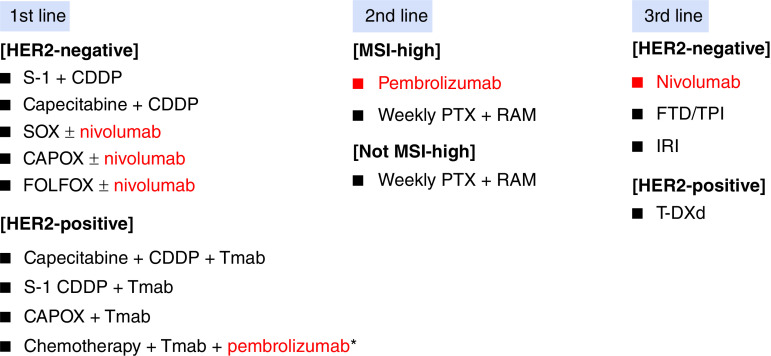
Recommended chemotherapy regimens for AGC. Recommended regimens are listed for each line of treatment in the Japanese guidelines. Red letters are ICI. CDDP: Cisplatin; CAPOX: Capecitabine + oxaliplatin; FOLFOX: 5-FU + leucovorin + oxaliplatin; IRI: Irinotecan; PTX: Paclitaxel; RAM: Ramucirumab; Tmab: Trastuzumab; FTD/TPI: Trifluridine/tipiracil; S-1: Tegafur/gimeracil/oteracil potassium; SOX: Tegafur/gimeracil/oteracil potassium + oxaliplatin; T-DXd: Trastuzumab deruxtecan. *This regimen is only available in USA.

In recent years, there has been remarkable research on ICI for GC, and many clinical trials have been conducted. However, anticancer drugs available for clinical use are still inadequate and limited (Figure 2).

To mention important regimens, nivo + chemotherapy is available for first-line treatment in the USA and Japan, not limited to CPS. However, it should be noted that the effect may be reduced in CPS <5. In addition, pembro + trastuzumab + chemotherapy is available for HER2-positive patients in USA. Next, patient selection is important for the use of pembrolizumab in second-line therapy. For example, it has been suggested that patients with ECOG PS0, CPS >10, and MSI-H are preferable. In the third line or later line, the result of ATTRACTION-2 showed of single-agent nivo, which is recommended for patients whose prior therapy did not include anti-PD-1 antibodies.

Recently, combination therapy with anti-PD-1 antibodies and other antibodies has emerged. Combination therapy with anti-CTLA4 antibodies and TKIs is expected to yield positive results. Other ICIs such as LAG3 antibodies, TIGIT, and BsAb are also emerging, and further investigation of their efficacy as well as their selection and AEs will be an issue.

## Future perspective

Great advances in the profiling of genomic, proteomic, and immunological approaches have been clarifying the immunological landscape, and, today, in addition to the mainstream combination of ICIs and chemotherapy as primary treatment for AGC, combining ICIs with molecular-targeted agents is also being assessed, and the range of treatment options is constantly expanding. Clinicians need to constantly update information and provide the best possible treatment.

Bearing in mind that only a fraction of patients experiences real clinical benefit from ICI treatments, developing new immunotherapies remains an urgent unmet need. To this end, improved biomarkers for selecting patients mostly likely to benefit from single-agent anti-PD-1 or PD-L1 inhibitors in earlier treatment lines or combination therapies with chemotherapy, another ICIs and molecular targeted drugs aimed at overcoming resistance should also be established in the near future. It is hoped that this will improve patient selection for ICIs and optimize patient response.

**Executive summary**

- Globally, gastric cancer is the fourth most common malignant tumor and also ranks fourth as a cause of death from cancer.
- For advanced gastric cancer (AGC), first-line treatment is generally systemic chemotherapy but the effectiveness of immune checkpoint inhibitors (ICIs) was established.

**The first-line setting**

- The KEYNOTE-062 phase III trial showed that pembro monotherapy was certainly not more effective than chemotherapy for tumors with CPS ≥1 but it did tend to prolong OS relative to chemotherapy when CPS was ≥10.
- The CheckMate-649 trial resulted in nivo + chemotherapy yielded significant improvements in OS and PFS regardless of tumor PD-L1 levels.

**The second-line setting**

- KEYNOTE-061, a phase III trial of second-line pembro for AGC with PD-L1 CPS ≥1 failed to show any significant prolongation of OS relative to paclitaxel.

**The third- & later-line setting**

- In the phase III ATTRACTION-2 trial, nivo treatment was reported to result in improved OS and PFS relative to placebo for AGC patients.
- The phase II trial KEYNOTE-059 showed the effectiveness and safety of pembro as a third-line treatment.

**The perioperative setting**

- According to the result of The phase II NEONIPIGA trial, neoadjuvant nivolumab + ipilimumab for localized MMR-D/MSI-H GC or esophagogastric adenocarcinomas showed pathological CR was nearly 60%.
- Many other clinical trials are underway in perioperative setting.

**Other setting**

- To evaluate the effectiveness of combination therapy with anti-PD-1 and anti-CTLA4 antibodies, multikinase inhibitors or HER2 targeted drug, many clinical trial are underway.
- In addition to PD-1 and CTLA-4, trials targeting LAG3 and TIGIT, as well as trials in BiTE therapy and CAR-T cells therapy are ongoing.
